# Feasibility and efficacy of gemcitabine and docetaxel combination chemotherapy for bone and soft tissue sarcomas: multi-institutional retrospective analysis of 134 patients

**DOI:** 10.1186/s12957-016-1059-2

**Published:** 2016-12-08

**Authors:** Kazuhiro Tanaka, Susumu Joyama, Hirokazu Chuman, Hiroaki Hiraga, Hideo Morioka, Hideki Yoshikawa, Masami Hosaka, Mitsuru Takahashi, Tadahiko Kubo, Hiroshi Hatano, Mitsunori Kaya, Junya Toguchida, Yoshihiro Nishida, Akihito Nagano, Hiroshi Tsumura, Yukihide Iwamoto

**Affiliations:** 1 Department of Orthopaedic Surgery, Oita University, Idaigaoka 1-1, Hasama, Yufu, Oita 879-5593 Japan; 2 Department of Orthopaedic Surgery, Osaka Medical Center, Osaka, 537-8511 Japan; 3 Department of Orthopaedic Surgery, National Cancer Center Hospital, Tokyo, 104-0045 Japan; 4 Department of Orthopaedic Surgery, Hokkaido Cancer Center, Sapporo, 003-0804 Japan; 5 Department of Orthopaedic Surgery, Keio University, Tokyo, 160-0016 Japan; 6 Department of Orthopaedic Surgery, Osaka University, Osaka, 565-0871 Japan; 7 Department of Orthopaedic Surgery, Tohoku University, Sendai, 980-8575 Japan; 8 Department of Orthopaedic Surgery, Shizuoka Cancer Center, Shizuoka, 411-0934 Japan; 9 Department of Orthopaedic Surgery, Hiroshima University, Hiroshima, 734-0037 Japan; 10 Department of Orthopaedic Surgery, Niigata Cancer Center Hospital, Niigata, 951-8133 Japan; 11 Department of Orthopaedic Surgery, Sapporo Medical University, Sapporo, 060-8556 Japan; 12 Department of Orthopaedic Surgery, Kyoto University, Kyoto, 606-8501 Japan; 13 Department of Orthopaedic Surgery, Nagoya University, Nagoya, 466-8550 Japan; 14 Department of Orthopaedic Surgery, Gifu University, Gifu, 501-1194 Japan; 15 Department of Orthopaedic Surgery, Kyushu University, Fukuoka, 812-8582 Japan; 16 Present address: Kyushu Rosai Hospital, Kitakyushu, 800-0296 Japan

**Keywords:** Gemcitabine, Docetaxel, Sarcoma, Adjuvant chemotherapy, Adverse events

## Abstract

**Background:**

Bone and soft tissue sarcomas (BSTS) are rare malignant tumors. Recently, the combination of gemcitabine and docetaxel (GD) was shown to have activity as second-line setting in BSTS. However, the efficacy as first-line and adjuvant settings and precise profiles of adverse events in Japanese patients are not known yet. In the present study, the feasibility and efficacy of GD in patients with BSTS were investigated.

**Methods:**

Patients with BSTS treated with GD in our institutions were retrospectively analyzed. Information regarding clinical features, adverse events, and outcome was collected and statistically studied. Factors related to survival were analyzed using log-rank test and Cox proportional hazard regression method.

**Results:**

A total of 134 patients were analyzed. GD was carried out as adjuvant setting in 9, first-line in 23, second-line in 56, and third-or-greater line in 46 patients. The response rate (RR) for all patients was 9.7%. RR for the patients treated as adjuvant or first-line setting was 18.8%, whereas that as second-or-greater line was 6.9%. The median progression-free survival (PFS) and overall survival (OS) of all patients were 4.8 (95% CI 3.5–6.1) and 16.4 (95% CI 9.8–22.9) months, respectively. Survival tended to be better in the patients treated as first-line than in those treated as second-or-greater line. Multivariate analysis demonstrated that history of prior chemotherapy (*p* = 0.046) and response to GD (*p* = 0.009) was significantly associated with PFS and OS, respectively. The leucopenia and neutropenia were the most frequent adverse events, and grade 3 or 4 leucopenia and neutropenia were observed in 69.4 and 72.4% of the patients. Grade 2 or 3 pneumonitis was observed in one (0.7%) and four (3.0%) patients, respectively. All the patients with pneumonitis had experienced prior chemotherapy and/or radiotherapy.

**Conclusions:**

GD used as both first- and second/later line is effective chemotherapy for a proportion of patients with advanced BSTS. Higher response rate and better outcome was achieved in chemotherapy-naïve patients. This regimen is associated with high incidence of severe hematological toxicity, as well as the risk of severe pneumonitis, especially in pre-treated patients. GD is promising for further analysis by phase III study for the patients with BSTS.

## Background

Bone and soft tissue sarcomas (BSTS) are very rare malignant tumors. BSTS account for approximately 1% of all malignancies. According to the Bone and Soft Tissue Tumor Registry reported by the Musculoskeletal Tumor Committee of the Japanese Orthopaedic Association, only 591 cases of bone sarcoma (BS) and 1509 cases of soft tissue sarcomas (STS) were registered in 2013 in Japan [1, 2]. Because of the rareness of BSTS, it is difficult to develop novel treatments for the tumors. Current standard chemotherapy for BSTS consists of old reagents such as doxorubicin (DOX) and ifosfamide (IFO) [3, 4]. DOX has remained a key drug for many years in the treatment of BSTS, and its response rate (RR) for sarcomas is approximately 25%. IFO is another key drug for BSTS with RR of approximately 30%. The combination of DOX with IFO has been shown to improve outcomes of the patients with localized STS [5], whereas the combination failed to show the improvement of prognosis of the patients with advanced STS [6].

Gemcitabine (GEM) is a fluorine-substituted pyrimidine analog, and is phosphorylated to the diphosphate and triphosphate metabolites. These active metabolites inhibit DNA synthesis and exhibit anti-tumor effects [7]. Docetaxel (DOC) has the activity to inhibit the depolymerization of microtubular bundles to free tubulin [8], resulting in the disruption of cell mitosis. The RR of GEM and DOC alone for sarcomas was reported to be approximately 3 and 0%, respectively, and each drug was inactive as single agent for BSTS [9, 10].

Recent studies have demonstrated the efficacy of the combination of GEM plus DOC (GD) [11–16]. GD regimen indicated the high response rates for the patients with advanced uterine leiomyosarcomas in both first-line [17] and second-line settings [18]. It has been also reported that GD exhibited higher response rates, progression-free survival (PFS), and overall survival (OS) than single-agent GEM in a randomized phase II trial for patients with advanced STS previously treated by up to three prior regimens [19]. The efficacy of GD as the second-line setting for advanced BSTS was also reported in the prospective and retrospective studies [20, 21]. However, only one study has been reported showing the effects of GD as adjuvant or first-line treatment on BSTS [22].

GD regimen is also known to be feasible and less toxic than DOX+IFO regimen [11–18]. Although pulmonary toxicity of both GEM and DOC should be noted, the combination of GEM and DOC would not increase the pulmonary toxicity [19]. On the other hand, it has been demonstrated that pulmonary toxicities by GD were severe among Japanese patients in a Japan Clinical Oncology Group (JCOG) trial. The clinical trial, JCOG0104, evaluated the efficacy of GD for non-small cell lung cancer as second-line setting and resulted in the early termination due to unexpected three treatment-related deaths with interstitial pneumonitis [23]. These observations suggest that the incidence of pulmonary toxicity might be high in Japanese patients. However, there is no study demonstrating the precise profiles of the adverse events of GD including the pulmonary toxicities in Japanese patients with BSTS.

In the present study, we analyzed the profiles of the adverse events and efficacy of GD, including as adjuvant and first-line settings, for 134 patients with BSTS treated in the institutions participating in the Bone and Soft Tissue Tumor Study Group (BSTTSG) of JCOG. This study is one of the largest series of the patients with BSTS treated by GD [11].

## Methods

We retrospectively reviewed the records of the institutions of JCOG BSTTSG participating in the present study from July 2002 to September 2014. A total of 134 patients suffered from BSTS and treated by GD regimen in our institutions participating in JCOG BSTTSG were enrolled in the present study. This study was approved by the Institutional Review Board at Oita University, and a waiver of informed consent was provided.

The GD regimen consisted of GEM administrated in day 1 and 8 and DOC administrated in day 8. Basically, GEM was given intravenously in 30 min in 100 ml saline, and DOC was given intravenously over 60 min in 250 ml saline with premedication of 16 mg/day of dexamethasone for 3 days. Patients were given therapeutic and second-line prophylactic granulocyte colony stimulating factor if they had grade 4 neutropenia or febrile neutropenia. The median dose of GEM was 890 mg/m^2^/day (range 490–1000 mg/m^2^/day) and that of DOC was 70 mg/m^2^ (range 42–100 mg/m^2^). The chemotherapy was repeated until disease progression or intolerance to the regimen. The indication of adjuvant chemotherapy for BSTS was basically as follows: histologically high-grade sarcomas, larger than 5 cm in maximum diameter without metastasis, and deep-seated in tumor location. The median number of cycles of GD was three (range 1–14 cycles) for all patients. The mean follow-up period for 56 surviving patients was 18 months (range 1–75 months), and that for 78 patients who died was 14 months (range 1–72 months).

Toxicity was evaluated according to the National Cancer Institute Common Terminology Criteria for Adverse Events (NCI CTCAE) version 3.0. The radiological evaluation of the response to the chemotherapy was assessed using Response Evaluation Criteria in Solid Tumor (RECIST) ver. 1.1. The progression-free survival (PFS) was defined as the time period from the day GD started until the day of the first evidence of disease progression or death. The overall survival (OS) was defined as the time period from the day GD started until the day of death or last follow-up. The PFS and OS were calculated using the Kaplan-Meier method. Differences in survivals were assessed by the log-rank test and Cox proportional hazard regression method. Differences were considered significant when *p* values were <0.05. Statistical analysis was done using IBM SPSS Statistics 22.0 software (IBM, Armonk, NY, USA).

## Results

### Patient characteristics

A total of 134 patients were analyzed in the present study (Table 1). The median age of the patients was 53 years old (range 10–78 years old) at the treatment by GD. The sites of primary lesions were soft tissues in 105 patients, and bones in 29 patients. The two most frequent histologic tumor types of STS were leiomyosarcoma (*n* = 33) and undifferentiated pleomorphic sarcoma (*n* = 18). The histologic tumor types of BS were osteosarcoma 15, Ewing sarcoma 3, leiomyosarcoma 5, undifferentiated pleomorphic sarcoma of bone 2, and other 4.

**Table 1:** Patient characteristics (*n* = 134)

Age	Median (years)	53
	Range	10–78
Bone tumor		29 (21.6%)
Primary site	Humerus	2 (1.5%)
	Spine	6 (4.5%)
	Femur	16 (11.9%)
	Tibia	3 (2.2%)
	Others	2 (1.5%)
Soft tissue tumor		105 (78.4%)
Primary site	Head and neck	4 (3.0%)
	Shoulder girdle	3 (2.2%)
	Upper arm	3 (2.2%)
	Forearm	6 (4.5%)
	Other upper extremity	5 (3.7%)
	Chest wall	4 (3.0%)
	Buttock	6 (4.5%)
	Retroperitoneum	14 (10.4%)
	Visceral	10 (7.5%)
	Thigh	25 (18.7%)
	Leg	9 (6.7%)
	Other lower extremity	9 (6.7%)
	Others	7 (5.2%)
Histological subtype		
	Leiomyosarcoma	38 (28.4%)
	Undifferentiated pleomorphic sarcoma	20 (14.9%)
	Osteosarcoma	17 (12.7%)
	Liposarcoma	9 (6.7%)
	Synovial sarcoma	7 (5.2%)
	Malignant peripheral nerve sheath tumor	7 (5.2%)
	Angiosarcoma	4 (3.0%)
	Ewing sarcoma	4 (3.0%)
	Epithelioid sarcoma	4 (3.0%)
	Rhabdomyosarcoma	3 (2.2%)
	Others	21 (15.7%)
Presentation status		
	Localized	9 (6.7%)
	Metastatic or locally advanced	125 (93.3%)
Prior chemotherapy regimen		
	DOX+IFO	38 (28.4%)
	DOX alone	21 (15.7%)
	IFO alone	18 (13.4%)
	IFO+VP16	12 (9.0%)
	IFO+CDBCA+VP16	11 (8.2%)
	DOX+CDDP	6 (4.5%)
	None	32 (23.9%)
Prior radiation		45 (33.6%)

Among 134 patients, the metastatic and/or locally advanced BSTS were observed in 125 patients, and GD was carried out as first-line setting in 23 patients, second-line in 56 patients, and third-or-greater line in 46 patients. On the other hand, GD regimen was carried out as adjuvant chemotherapy with resection of localize primary tumor in 9 patients. The frequent chemotherapy regimens prior to GD for the patients with advanced BSTS were DOX+IFO (38 patients), DOX alone (21 patients), and IFO alone (18 patients). The median number of lines of prior chemotherapy was 1 (range 1–5 regimens). The prior radiation therapy had been carried out for 45 patients. Median dose of radiation was 56 Gy.

The median doses of GEM and DOC were 890 mg/m^2^/day (range 490–1000 mg/m^2^/day) and 70 mg/m^2^ (range 42–100 mg/m^2^) for the patients with advanced BSTS, and 900 mg/m^2^/day (range 550–900 mg/m^2^/day) and 70 mg/m^2^ (range 50–100 mg/m^2^) for the non-advanced cases. The median number of cycles of GD was 3 (range 1–14 cycles) for the advanced cases, and 5 (range 2–9 cycles) for the non-advanced cases treated as adjuvant chemotherapy. The two most frequent reasons for the discontinuation of GD regimen were progression of disease (81%) and adverse events (10%).

### Response and survival

The RECIST-assessed response rate was 9.7% (13/134) for all patients. Two patients were assessed as complete response (CR), 11 partial response (PR), 55 stable disease (SD), 55 progressive disease (PD), and 11 not evaluable (NE). The patients who had no prior chemotherapy, i.e., treated as adjuvant or first-line setting exhibited 1 CR and 5 PR, and RR was 18.8% (6/32). On the other hand, the patients treated as second-or-greater line had 1 CR and 6 PR, resulting in RR of 6.9% (7/102). Although the difference was not significant, RR of GD as adjuvant or first-line setting chemotherapy tended to be better than that for second-or-greater line setting chemotherapy. RR for the patients with BS was only 3.4% (1PR/29), whereas that for STS was 11.4% (2CR+10PR/105). Objective response by histological subtypes of BSTS was summarized in Table 2. The response (CR/PR) was observed in the patients with leiomyosarcoma, undifferentiated pleomorphic sarcoma, malignant peripheral nerve sheath tumor, angiosarcoma, and Ewing sarcoma. RR for leiomyosarcoma and undifferentiated pleomorphic sarcoma was 13.2% (5/38) and 15.0% (3/20), respectively (Table 2).

**Table 2:** Response to GD by histological subtypes

	CR	PR	SD	PD	NE	RR (%)
All	2	11	55	55	11	9.7
Leiomyosarcoma	0	5	19	12	2	13.2
Undifferentiated pleomorphic sarcoma	1	2	11	4	2	15.0
Osteosarcoma	0	0	7	8	2	0
Liposarcoma	0	0	2	5	2	0
Synovial sarcoma	0	0	3	4	0	0
Malignant peripheral nerve sheath tumor	0	1	0	5	1	14.3
Angiosarcoma	0	1	1	1	1	25.0
Ewing sarcoma	0	1	1	2	0	25.0
Epithelioid sarcoma	0	0	1	3	0	0
Rhabdomyosarcoma	0	0	2	1	0	0
Others	1	1	8	10	1	9.5

*CR* complete response, *PR* partial response, *SD* stable disease, *PD* progressive disease, *NE* not evaluable, *RR* response rate

**Fig. 1:**
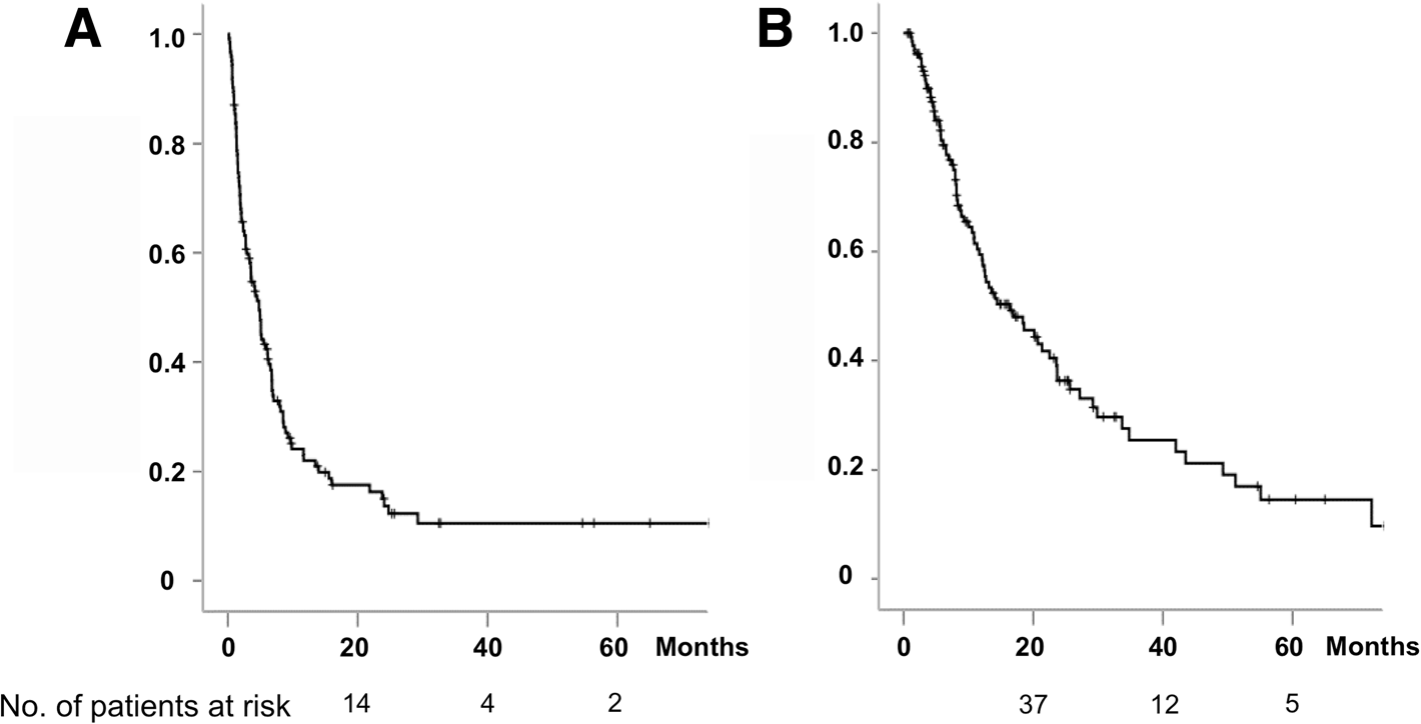
Kaplan-Meier estimates of survival of all patients. **a** Progression-free survival. **b** Overall survival

At the last follow-up time, 56 patients were alive and 78 patients were dead. The median PFS for all patients was 4.8 months (95% CI 3.5–6.1) (Fig. 1a). The median PFS for the patients treated by GD as adjuvant therapy (*n* = 9) was not reached yet (*p* < 0.001), and estimated 5-year PFS was 70%. The median PFS as first-line (*n* = 23) was 6.7 months (95% CI 5.9–7.5), that as second-line (*n* = 56) was 4.0 months (95% CI 2.5–5.6), and that as third-or-greater line (*n* = 46) was 2.0 months (95% CI 0.2–3.9), respectively.

**Fig. 2:**
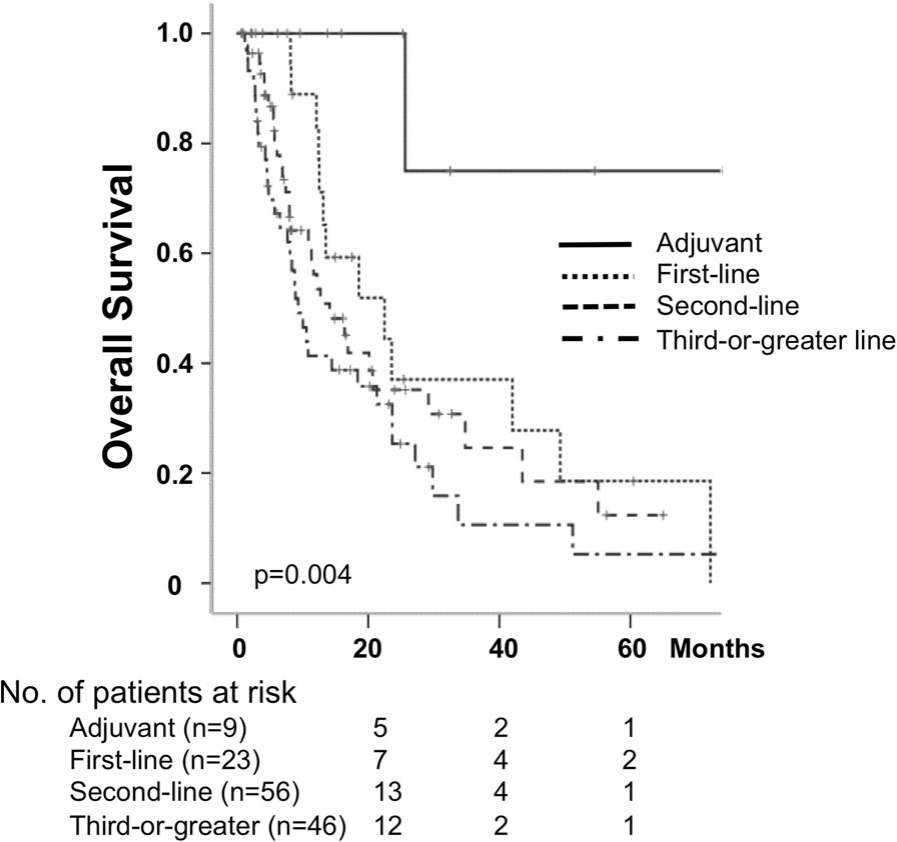
Kaplan-Meier estimates of overall survival of the patients treated with GD as adjuvant, first-line, second-line, and third-or-greater line setting

The median OS of all patients was 16.4 months (95% CI 9.8–22.9) (Fig. 1b). The median OS for the patients treated by GD as adjuvant therapy was not reached yet (*p* = 0.004), and estimated 5-year OS was 75%. The median OS as first-line was 22.5 months (95% CI 7.4–37.6), that as second-line was 14.1 months (95% CI 8.5–19.7), and that as third-or-greater line was 9.3 months (95% CI 6.6–12.0), respectively. Although the difference in OS between first-line and second-or-greater line setting was not significant, survival tended to be better in the patients treated as first-line setting (Fig. 2).

Next, the effects of doses on GD were investigated. The patients treated with GEM 900 mg/m^2^/day and DOC 70 mg/m^2^ (G900/D70) (*n* = 18), which was the same as the median dose, exhibited 1 CR and 2 PR, and RR rate was 16.7%. The patients treated with doses higher than G900/D70 (>G900/D70) (the mean dose of GEM 939 mg/m^2^/day and DOC 89 mg/m^2^) (*n* = 32) exhibited only 1 PR and RR was 3.1%. The median PFS and OS of patients with G900/D70 were 7.2 months (0.4–32.5 months) and 13.7 months (0.6–32.5 months), respectively, whereas those with >G900/D70 were 3.3 months (0.3–54.6 months) and 6.3 months (1.6–54.6 months), respectively.

Univariate analysis for the potential prognostic factors for PFS in 134 patients was carried out (Table 3). Histologic subtype (undifferentiated pleomorphic sarcoma vs. leiomyosarcoma vs. others) and history of prior chemotherapy (first-line setting vs. second-or-greater line setting) were significantly correlated with PFS. Age, the sites of primary lesions, bone or soft tissue tumors, response to GD (CR or PR vs. SD or PD), and doses of GD were not prognostic factors for PFS. Multivariate analysis also performed to demonstrate factors influencing to PFS. History of prior chemotherapy (*p* = 0.046) was a significant prognostic factor for PFS (Table 3).

**Table 3:** Univariate and multivariate analyses for progression-free survival

	Univariate analysis	
Factors	HR (95% CI)	*p* value
Age (years)		
<50 (vs. ≥50)	1.23 (0.82–1.84)	0.322
Tumor origin		
Bone (vs. soft tissue)	1.05 (0.65–1.69)	0.843
Primary site		0.648
Trunk (vs. extremities)	1.20 (0.67–2.15)	0.542
Retroperitoneum (vs. extremities)	1.02 (0.56–1.85)	0.957
Visceral (vs. extremities)	1.48 (0.78–2.83)	0.231
Histological subtype		0.008
UPS (vs. leiomyosarcoma)	1.20 (0.61–2.35)	0.591
Others (vs. leiomyosarcoma)	2.01 (1.23–3.21)	0.004
Prior chemotherapy		0.028
Second-line (vs. first-line)	0.99 (0.55–1.78)	0.974
Third-or-greater line (vs. first-line)	1.74 (0.97–3.12)	0.064
Response to GD		
SD/PD (vs. CR/PR)	2.05 (0.99–4.23)	0.053
	Multivariate analysis	
Factors	HR (95% CI)	*p* value
Age (years)		
≥50 (vs. <50)		
Tumor origin		
Bone (vs. soft tissue)		
Primary site		
Trunk (vs. extremities)		
Retroperitoneum (vs. extremities)		
Visceral (vs. extremities)		
Histological subtype		
UPS (vs. leiomyosarcoma)		
Others (vs. leiomyosarcoma)		
Prior chemotherapy		0.043
Second-line (vs. first-line)	1.02 (0.56–1.83)	0.974
Third-or-greater line (vs. first-line)	1.71 (0.95–3.07)	0.074
Response to GD		
SD/PD (vs. CR/PR)		

On the other hand, histologic subtype (*p* = 0.002) and response to GD (*p* = 0.010) were significant prognostic factors for OS in univariate analysis (Table 4). Multivariate analysis demonstrated that response to GD (*p* = 0.009) was significantly associated with OS (Table 4). The patients with leiomyosarcoma and UPS showed similar OS and PFS; however, the prognosis of leiomyosarcoma patients was significantly better than that of other histologic subtypes excluding UPS (for PFS, *p* = 0.004 and for OS, *p* = 0.001).

**Table 4:** Univariate and multivariate analyses for overall survival

	Univariate analysis	
Factors	HR (95% CI)	*p* value
Age (years)		
<50 (vs. ≥50)	1.20 (0.76–1.89)	0.436
Tumor origin		
Bone (vs. soft tissue)	1.32 (0.77–2.27)	0.318
Primary site		0.633
Trunk (vs. extremities)	0.85 (0.43–1.68)	0.639
Retroperitoneum (vs. extremities)	0.65 (0.31–1.37)	0.259
Visceral (vs. extremities)	1.15 (0.56–2.35)	0.702
Histological subtype		0.002
UPS (vs. leiomyosarcoma)	1.24 (0.55–2.81)	0.608
Others (vs. leiomyosarcoma)	2.54 (1.46–4.42)	0.001
Prior chemotherapy		0.126
Second-line (vs. first-line)	1.38 (0.72–2.65)	0.332
Third-or-greater line (vs. first-line)	1.90 (1.00–3.62)	0.052
Response to GD		
SD/PD (vs. CR/PR)	3.81 (1.37–10.55)	0.010
	Multivariate analysis	
Factors	HR (95% CI)	*p* value
Age (years)		
≥50 (vs. <50)		
Tumor origin		
Bone (vs. soft tissue)		
Primary site		
Trunk (vs. extremities)		
Retroperitoneum (vs. extremities)		
Visceral (vs. extremities)		
Histological subtype		
UPS (vs. leiomyosarcoma)		
Others (vs. leiomyosarcoma)		
Prior chemotherapy		
Second-line (vs. first-line)		
Third-or-greater line (vs. first-line)		
Response to GD		
SD/PD (vs. CR/PR)	3.99 (1.42–11.22)	0.009

### Adverse events

There was no treatment-related death. The leucopenia and neutropenia were the most frequent adverse events by GD for BSTS (Table 5). Grade 3 or 4 leucopenia and neutropenia were observed in 93 (69.4%) and 97 (72.4%) patients. Grade 3 or 4 anemia and thrombocytopenia were observed in 25 (18.7%) and 37 (27.6%) patients.

**Table 5:** Adverse events in all patients (*n* = 134)

Adverse event	G0	G1	G2	G3	G4	NE	All grades (%)	Grade 3/4 (%)
Leucocyte	9	8	21	44	49	3	91.0	69.4
Neutrophils	9	7	17	30	67	4	90.3	72.4
Hemoglobin	27	41	38	23	2	3	77.6	18.7
Platelets	55	21	18	24	13	3	56.7	27.6
AST	87	38	3	3	0	3	32.8	2.2
ALT	82	35	9	5	0	3	36.6	3.7
Anorexia	52	59	15	2	0	6	56.7	1.5
Nausea	65	49	14	1	0	5	47.8	0.7
Vomiting	103	22	4	0	0	5	19.4	0.0
Diarrhea	112	13	2	2	0	5	12.7	1.5
Fatigue	50	59	15	6	0	4	59.7	4.5
Febrile neutropenia	120	0	0	13	0	1	9.7	9.7
Fever	97	22	12	1	0	2	26.1	0.7
Infection	118	4	5	5	1	1	11.2	4.5
Dyspnea	124	4	1	4	0	1	6.7	3.0
Interstitial pneumonia	124	4	1	4	0	1	6.7	3.0
Neuropathy	128	0	5	0	0	1	3.7	0.0
Rash	114	6	8	5	0	1	14.2	3.7
Edema	131	2	1	0	0	0	2.2	0.0
Mucositis	130	1	3	0	0	0	3.0	0.0
Allergic reaction	132	0	2	0	0	0	1.5	0.0

*AST* aspartate transaminase, *ALT* alanine aminotransferase

Febrile neutropenia was observed in 13 out of 134 patients (9.7%). The most frequent non-hematological toxicities were nausea and anorexia; however, these adverse events were modest. Grade 3 nausea and anorexia were observed only in two (1.5%) and one (0.7%) patients. Grade 4 non-hematological toxicity was found only in one patient (0.7%) as infection. Regarding lung toxicities, dyspnea and pneumonitis were observed in nine patients (6.7%) as all grades. Grade 2 or 3 pneumonitis was observed in one (0.7%) and four (3.0%) patients, respectively. All the five patients were assessed as interstitial pneumonitis and successfully treated by steroid-pulse therapy. All the patients with pneumonitis had experienced previous chemotherapy. Four out of five patients received GD as third-or-greater line and one as second-line chemotherapy. Three out of five patients with grade 2 or 3 pneumonitis had been treated by prior radiotherapy, and dose and site of radiation were 30 Gy to the chest wall, 54 Gy to the lung, and 50 Gy to the thigh.

Among 32 patients treated by GD as adjuvant or first-line therapy, grade 3 or 4 leucopenia and neutropenia were observed in 17 (53.1%) and 21 (65.6%) patients (Table 6). Grade 3 or 4 anemia and thrombocytopenia were observed in five (15.6%) and three (9.4%) patients. Grade 4 non-hematological toxicity was not observed. Grade 3 nausea and anorexia were not observed, whereas grade 3 dyspnea was observed in one patient (3.1%). The pneumonitis was observed in two patients (6.3%) only as grade 1.

**Table 6:** Adverse events in patients treated as adjuvant/first-line setting (*n* = 32)

Adverse event	G0	G1	G2	G3	G4	NE	All grades (%)	Grade 3/4 (%)
Leucocyte	3	3	8	10	7	1	87.5	53.1
Neutrophils	2	3	5	8	13	1	90.6	65.6
Hemoglobin	10	8	8	5	0	1	65.6	15.6
Platelets	19	3	6	3	0	1	37.5	9.4
AST	22	6	2	1	0	1	28.1	3.1
ALT	22	5	1	3	0	1	28.1	9.4
Anorexia	13	14	5	0	0	0	59.4	0.0
Nausea	17	11	4	0	0	0	46.9	0.0
Vomiting	26	5	1	0	0	0	18.8	0.0
Diarrhea	26	4	1	1	0	0	18.8	3.1
Fatigue	14	12	5	1	0	0	56.3	3.1
Febrile neutropenia	29	0	0	3	0	0	9.4	9.4
Fever	24	6	1	1	0	0	25.0	3.1
Infection	29	0	1	2	0	0	9.4	6.3
Dyspnea	31	0	0	1	0	0	3.1	3.1
Interstitial pneumonia	30	2	0	0	0	0	6.3	0.0
Neuropathy	32	0	0	0	0	0	0.0	0.0
Rash	24	4	3	1	0	0	25.0	3.1
Edema	30	2	0	0	0	0	6.3	0.0
Mucositis	31	0	1	0	0	0	3.1	0.0
Allergic reaction	32	0	0	0	0	0	0.0	0.0

*AST* aspartate transaminase, *ALT* alanine aminotransferase

For the patients treated as second-or-greater line (*n* = 102), grade 3 or 4 leucopenia and neutropenia were observed in both 76 patients (74.5%) (Table 7). Grade 3 or 4 anemia and thrombocytopenia were observed in 20 (19.6%) and 34 (33.3%) patients. The lung toxicities were observed as dyspnea and pneumonitis in eight (7.8%) and seven (6.9%) patients as all grades, respectively. Grade 3 pneumonitis was observed in four patients (3.9%). The incidence of grade 3 and 4 leucopenia (HR 0.214, 95% CI 0.021–0.406, *p* = 0.022) and thrombocytopenia (HR 0.24, 95% CI 0.103–0.376, *p* = 0.008) of the patients treated as adjuvant or first-line setting were significantly less frequent than those as second-or-greater line setting.

**Table 7:** Adverse events in patients treated as second-or-greater line setting (*n* = 102)

Adverse event	G0	G1	G2	G3	G4	NE	All grades (%)	Grade 3/4 (%)
Leucocyte	6	5	13	34	42	2	92.2	74.5
Neutrophils	7	4	12	22	54	3	90.2	74.5
Hemoglobin	17	33	30	18	2	2	81.4	19.6
Platelets	36	18	12	21	13	2	62.7	33.3
AST	65	32	1	2	0	2	34.3	2.0
ALT	60	30	8	2	0	2	39.2	2.0
Anorexia	39	45	10	2	0	6	55.9	2.0
Nausea	48	38	10	1	0	5	48.0	1.0
Vomiting	77	17	3	0	0	5	19.6	0.0
Diarrhea	86	9	1	1	0	5	10.8	1.0
Fatigue	36	47	10	5	0	4	60.8	4.9
Febrile neutropenia	91	0	0	10	0	1	9.8	9.8
Fever	73	16	11	0	0	2	26.5	0.0
Infection	89	4	4	3	1	1	11.8	3.9
Dyspnea	93	4	1	3	0	1	7.8	2.9
Interstitial pneumonia	94	2	1	4	0	1	6.9	3.9
Neuropathy	96	0	5	0	0	1	4.9	0.0
Rash	90	2	5	4	0	1	10.8	3.9
Edema	101	0	1	0	0	0	1.0	0.0
Mucositis	99	1	2	0	0	0	2.9	0.0
Allergic reaction	100	0	2	0	0	0	2.0	0.0

*AST* aspartate transaminase, *ALT* alanine aminotransferase

## Discussion

Recent studies have demonstrated that the combination of GEM with DOC is effective for BSTS, and that GD regimen is supposed to have milder toxicity than DOX+IFO, the standard regimen for STS [11–16, 19]. In a clinical trial JCOG0304, in which DOX+IFO was administrated for high-grade STS, the incidences of grade 3 and 4 leucopenia, neutropenia, anemia, thrombocytopenia, and febrile neutropenia were 97.2, 98.6, 55.6, 15.3, and 18.2%, respectively [24, 25]. It has been reported that the incidences of grade 3 and 4 toxicities observed in GD were lower than those in DOX+IFO; leucopenia approximately 10–40%, neutropenia 10–70%, anemia 5–15%, thrombocytopenia 10–40%, and febrile neutropenia 0–10% [11–16, 19]. In the present study, the incidences of grade 3 and 4 adverse events by GD were consistent with the previous studies. Grade 3 or 4 neutropenia, anemia, and febrile neutropenia were observed in 72.4, 18.7, and 9.7%, respectively, of the patients received GD. Furthermore, when GD was administrated as adjuvant or first-line setting, the incidences of grade 3 or 4 leucopenia and thrombocytopenia were significantly lower than those as second-or-greater setting.

On the other hand, GD regimen is also known to have the risk of interstitial pneumonitis. In a randomized trial, JCOG0104, which was conducted to confirm the superiority of GD on survival of the patients with previously treated non-small cell lung cancer (NSCLC) over DOC alone, was early terminated due to the unexpectedly high incidence of interstitial pneumonia (grade 3 and 4 pneumonitis was observed in 12.3%) and three treatment-related deaths (4.6%) in the GD arm [23]. The risk of lung toxicities by GD could not be ignored especially in Japan, since the unexpected severe pneumonitis in JCOG0104 was observed in Japanese patients.

Since few interstitial pneumonia were described in the past studies of GD for BSTS [11–16, 19–22], it has been supposed that GD would be relatively safe regarding interstitial pneumonia in the treatment of BSTS. In the present study, interstitial pneumonia was observed 9 (6.7%) out of 134 patients for all grade and 4 (3.0%) for grade 3. All the patients (five out of 134) with symptomatic pneumonia (grade 2/3) were successfully treated by steroid-pulse therapy, and no regimen-related death was observed. It was noteworthy that all five patients were received prior chemotherapy, and median number of previous regimens was 3. Furthermore, three out of five patients had been treated with radiotherapy. When GD was given as first-line or adjuvant setting, symptomatic pneumonitis was not observed. Although definite risk factors for interstitial pneumonia caused by GD are still unknown, a careful attention to lung toxicity should be paid even in the treatment for BSTS, especially when the patient has been treated with prior chemotherapy and/or radiotherapy.

The feasibility and efficacy of GD for BSTS have been previously reported from the USA and Europe [11, 12, 16, 19–22]; however, those were not well analyzed in Asian population, especially in Japan. Only a few small studies of GD for BSTS were reported from Asian countries [13–15]. Furthermore, there was no description about interstitial pneumonia or lung toxicities by GD in these studies. Thus, the present study is the largest series of GD for Asian patients with BSTS, and is reporting the lung toxicities of GD in the treatment for BSTS in Asian patients for the first time.

In addition to the less toxicity, GD regimen is also thought to be as effective as DOX+IFO. In a randomized phase II trial comparing perioperative chemotherapy with GD and DOX+IFO for STS, 2-year PFS in the GD arm and the DOX+IFO arm were 74 and 57%, respectively [22]. The results suggested that GD is promising for a phase III trial.

In the present study, the results demonstrated promising effects of GD especially as adjuvant or first-line setting on survival of the patients with BSTS. Taken together, the JCOG BSTTSG is now conducting a randomized phase II/III study, JCOG1306, to elucidate the efficacy and safety of perioperative chemotherapy by GD comparing with DOX+IFO for operable high-grade STS [26].

It is noteworthy that the dose of GD and its efficacy were not paralleled in the present study. RR and survival in the patients treated with G900/D70 were better than those with >G900/D70. One possible explanation of the discrepancy was the influence of adverse effects caused by GD. The incidences of adverse events in the patients treated with >G900/D70 were more frequent than those with G900/D70. For instance, neutropenia and decrease in platelet were observed in 83.3 and 38.9% of the patients in G900/D70 group, whereas those in >G900/D70 group were 96.9 and 62.5%, respectively. Thus, it is possible that the higher incidence of adverse events in >G900/D70 group might associate with the inferior outcome because of discontinuation or dose reduction of GD. It has been reported that approximately half of the patients were required dose reduction of GD because of adverse effects in the previous randomized phase II trials using G900/D100 [18, 21]. Thus, such doses around G900/D100 might be too high for the patients with BSTS.

Another possibility was the influence of previous treatments. Since the number of chemotherapy-naïve patients was larger in G900/D70 group (50.0% of the patients) than that in >G900/D70 group (31.3%), there is a possibility that more drug-resistant tumors were included in the latter group, which might lead to the lower response to GD. In this regard, when the response to GD was compared only for the chemotherapy-naïve patients, RR and median OS were 0% (0/10) and 16.2 months in >G900/D70 group, whereas those were 33.3% (3/9) and 13.8 months comparably in G900/D70 group, respectively. These results suggest that G900/D70 might be useful for BSTS, especially in the treatment for chemotherapy-naïve patients.

In summary, it is suggested that the incidences of the severe adverse events including lung toxicities in the patients without prior chemotherapy or radiotherapy were lower than those with prior chemotherapy and/or radiation. GD might be effective not only as second-line therapy for advanced BSTS but also as adjuvant or first-line chemotherapy for BSTS. Since GD can be administered in an outpatient setting due to its lower toxicities, GD is promising for further investigation by phase III trials JCOG1306 for the patients with BSTS.

## Conclusions

This is the first report demonstrating the precise profiles of the adverse events of GD for the Japanese patients with BSTS, and one of the largest series analyzing 134 patients with BSTS treated by GD. GD used as both first- and second/later line is effective chemotherapy for a proportion of patients with advanced BSTS. Higher response rate and better outcome were achieved in chemotherapy-naïve patients. This regimen is associated with high incidence of severe hematological toxicity, as well as the risk of severe pneumonitis, especially in pre-treated patients.

## Abbreviations

BS: Bone sarcomas
BSTS: Bone and soft tissue sarcomas
BSTTSG: Bone and Soft Tissue Tumor Study Group
CR: Complete response
DOC: Docetaxel
DOX: Doxorubicin
GD: Gemcitabine and docetaxel
GEM: Gemcitabine
IFO: Ifosfamide
JCOG: Japan Clinical Oncology Group
NE: Not evaluable
OS: Overall survival
PD: Progressive disease
PFS: Progression-free survival
PR: Partial response
RECIST: Response Evaluation Criteria in Solid Tumor
RR: Response rate
SD: Stable disease
STS: Soft tissue sarcomas
